# New insight into chondroitin and heparosan-like capsular polysaccharide synthesis by profiling of the nucleotide sugar precursors

**DOI:** 10.1042/BSR20160548

**Published:** 2017-02-20

**Authors:** Odile Francesca Restaino, Irene di Lauro, Rosaria Di Nuzzo, Mario De Rosa, Chiara Schiraldi

**Affiliations:** Department of Experimental Medicine, Section of Biotechnology and Molecular Biology, University of Campania Luigi Vanvitelli, ex Second University of Naples, Via De Crecchio 7, 80138 Naples, Italy

**Keywords:** Capillary electrophoresis, capsular polysaccharides, chondroitin, heparosan, nucleotide sugars

## Abstract

*Escherichia coli* K4 and K5 capsular polysaccharides (K4 and K5 CPSs) have been used as starting material for the biotechnological production of chondroitin sulfate (CS) and heparin (HP) respectively. The CPS covers the outer cell wall but in late exponential or stationary growth phase it is released in the surrounding medium. The released CPS concentration was used, so far, as the only marker to connect the strain production ability to the different cultivation conditions employed. Determining also the intracellular UDP-sugar precursor concentration variations, during the bacterial growth, and correlating it with the total CPS production (as sum of the inner and the released ones), could help to better understand the chain biosynthetic mechanism and its bottlenecks. In the present study, for the first time, a new capillary electrophoresis method was set up to simultaneously analyse the UDP-glucose (UDP-Glc), UDP-galactose (UDP-Gal), UDP-*N*-acetylgalactosamine (UDP-GalNAc), UDP-*N*-acetylglucosamine (UDP-GlcNAc) and UDP-glucuronic acid (UDP-GlcA) and the inner CPS portion, extracted at the same time from the bacterial biomasses; separation was performed at 18°C and 18 kV with a borate-based buffer and detection at 200 nm. The *E. coli* K4 and K5 UDP-sugar pools were profiled, for the first time, at different time points of shake flask growths on a glycerol-containing medium and on the same medium supplemented with the monosaccharide precursors of the CPSs: their concentrations varied from 0.25 to 11 μM·g_cdw_^−1^, according to strain, the type of precursor, the growth phase and the cultivation conditions and their availability dramatically influenced the total CPS produced.

## Introduction

**Figure 1:**
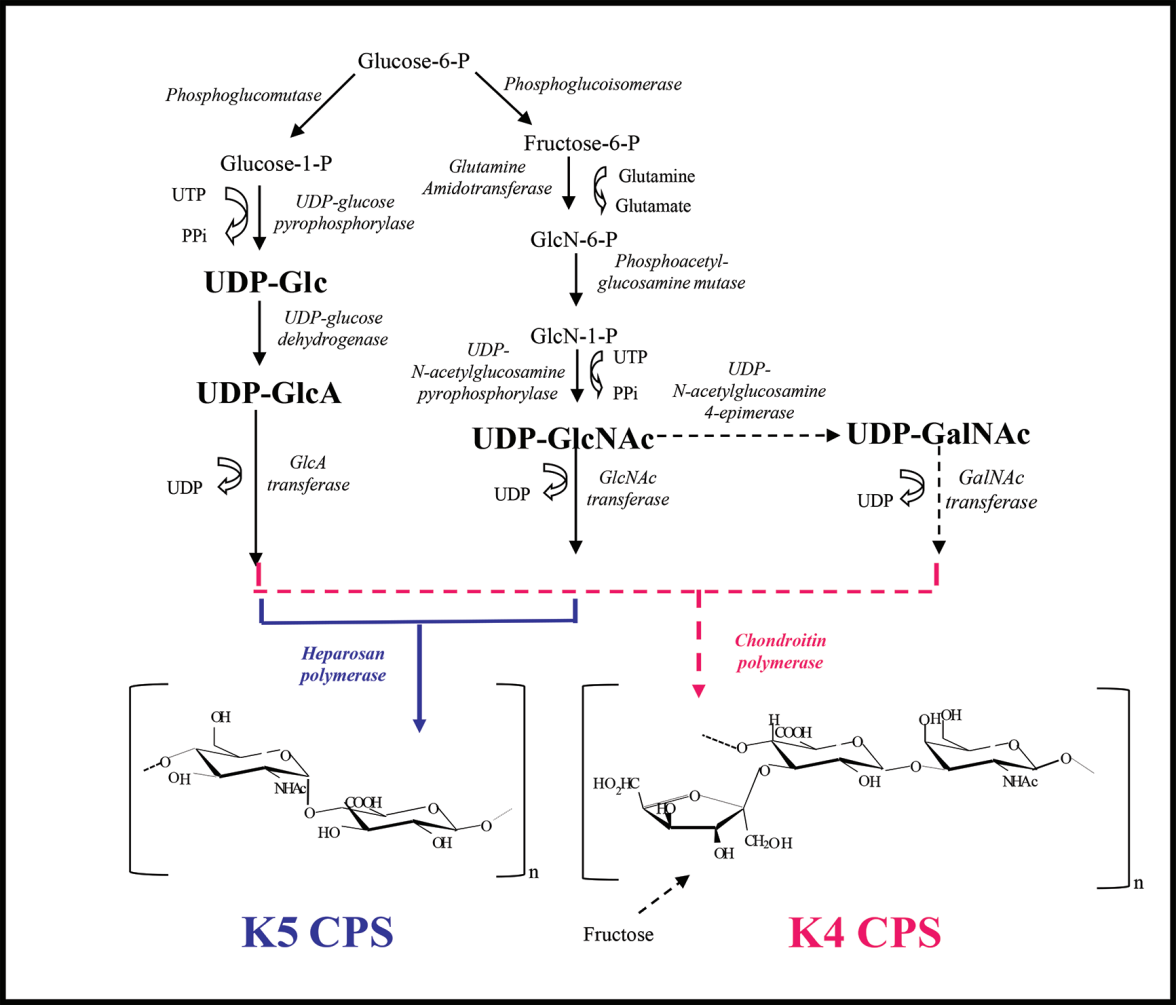
*E. coli* K4 and K5 pathways of UDP-sugar and CPS synthesis

*Escherichia coli* O5:K4:H4 and O10:K5:H4 capsular polysaccharides (K4 and K5 CPSs) have chondroitin and heparosan-like structures respectively, being made of [→4)-β-D-Glc*p*A-(1→3)-β-D-Gal*p*NAc-(1→]_n_, with an extra fructose monosaccharide bounded to the glucuronic acid (GlcA) residue, and of [→4)-β-D-Glc*p*A-(1→4)-α-D-Glc*p*NAc-(1→]_n_, correspondently [1,2]. Thanks to this structural similarity, these CPSs have been considered, in the last years, good starting materials for the biotechnological production of chondroitin sulfate (CS) and heparin (HP), the two mammalian glycosaminoglycans (GAGs) widely used as active principles of anti-osteoarthritis and anti-thrombic drugs [3,4]. To obtain natural-like molecules, the design of the biotechnology processes coupled fermentative-based production and purification procedures with either chemosynthetic or chemoenzymatic modification and sulfation steps [5–11]. The achievement of high titres of CPSs, during the fermentation phase, is considered a key factor to have economical advantageous yields and cost-effective productions [6,12]. Previous studies demonstrated that the CPS production is dependent on both growth and metabolic conditions and different approaches have been used so far to enhance the natural bacterial CPS production aptitude by optimizing the physiological growth conditions and the nutrient requirements [5,6,13,14], by exploiting high cell density fermentation strategies [6,12] or by employing molecular biology tools to obtain new over-producing strains [15–19]. In one of these studies, a 68% increase of the K4 CPS concentration at 24 h was obtained by adding to the medium, at the beginning of the growth, the two monosaccharides constituting the capsular chain, the *N*-acetylgalactosamine (GalNAc) and the GlcA; thus, it can be argued that the polysaccharide synthesis may be limited by unsufficient amounts of precursors inside the bacterial cells [14]. In *E. coli* K4 and K5, the CPS biosynthesis is performed in the cytoplasm by glycosyltransferase enzymes that extend progressively the nascent chain by addition of UDP-sugars to the non-reducing end of the chain. For *E. coli* K4 CPS synthesis, studies demonstrated that the fructose residue is not involved in the main elongation process performed by the glycosyltransferase enzymes but it is added only in a second moment, as branch, after the chain assembly [20]. The UDP precursors are synthesized from the carbon sources in the medium according to two specific pathways that in the two strains are similar: one way brings to the UDP-glucuronic acid formation (UDP-GlcA), passing through the UDP-glucose intermediate (UDP-Glc), whereas the other way drives to the UDP-*N*-acetylglucosamine formation (UDP-GlcNAc), which in *E. coli* K4 is then further converted into UDP-*N*-acetylgalactosamine (UDP-GalNAc) (Figure 1) [14,21]. Once synthesized the CPS is moved outside the wall to cover the cell [22] but, during the bacterial growth, in particular in the late exponential or stationary phase, the CPS is released in the surrounding medium [5,6]. Although the metabolic pathways that drive to the synthesis of K4 and K5 CPSs are known [14,21], no information is reported in literature on the concentrations of UDP-sugars available inside the cells during the cell growth, as well as on their variations. Profiling the nucleotide sugar pools could provide a metabolic fingerprint for both wild-type and recombinant strains, also in different growth conditions, it could help to understand and eventually sort out the bottlenecks in the synthetic pathway (like a limiting lack of precursors or an unbalanced ratio between them) and could provide useful information for designing new over-producing strains and/or to improve CPS production yields also by employing tailored-cut fermentation strategies. So far only a few methods have been described in literature for the UDP-sugar determination by capillary electrophoresis and, mainly, as intermediates extracted from animal tissues [23,24]; in particular the separation of UDP-Glc, UDP-galactose (UDP-Gal), UDP-GalNAc and UDP-GlcNAc, but not of UDP-GlcA, was reported by using borate-based buffers [23,24]. One of this method was slightly modified in a previous study to contemporary analyse UDP-Glc, UDP-GalNAc and UDP-GlcA as extracted from a recombinant *E. coli* K4 strain at only two time points of the growth [17], but it was not used to separate a mixture containing also UDP-GlcNAc; if the peaks of two amino nucleotide sugar peaks are not separated and overlapped that could result critical for an exact quantification of the UDP pools. Besides, in that previous study, the possibility to simultaneously determine the inner (internal or cell-bounded) CPS amount, extracted from the cells together with the UDP-sugars, was not taken in consideration. Developing a good analytical tool to determine the UDP-sugar concentrations and correlate their availability with the kinetic of total CPS production, as sum of the released and inner portion, during the bacterial growth could instead give additional information on the biosynthetic mechanism and how it works in different growth conditions. To address this issue in the present study, a new high-performance capillary electrophoresis (HPCE) method was set up and optimized for the contemporary analysis of UDP-Glc, UDP-Gal, UDP-GalNAc, UDP-GlcNAc, UDP-GlcA and of the inner CPSs as extracted from *E. coli* K4 and K5 wild-type cells; the nucleotide sugar concentrations were then evaluated at different time points of shake flask growths in two different conditions, on a glycerol-based medium and on the same medium supplemented with the CPS monosaccharide precursors [14]. The determined inner portion of CPS was summed with the released one in order to obtain the total amount produced at different time points of the bacterial growth and to correlate it with the UDP-sugar precursor availability in the different growth conditions.

## Materials and methods

### Materials

The standards and reagents used in the HPCE analyses were from Sigma–Aldrich (St. Louis, MO, USA), as well as the glycerol and the salts used for the growth medium, whereas soya peptone was from Oxoid (United Kingdom). The methanol used as a solvent for the biomass extraction was from Carlo Erba (Italy).

### Bacterial strain, microbial growths and biomass extraction

*E. coli* O5:K4:H4 and O10:K5:H4 were purchased from the Culture Collection, University of Göteborg, Sweden, both strains were stored in 20% (v/v) glycerol stock solutions and propagated as already described [5]. Growth experiments were performed in 1-litre shake flasks containing 200 ml of a glycerol and soya peptone medium [5], or on the same medium supplemented with 0.385 mM GalNAc and GlcA for *E. coli* K4 (14) or 0.385 mM GlcNAc and GlcA for *E. coli* K5, at pH 7.5, 37°C and 200 rpm in a rotary air shaker (Minitron, Infors, Switzerland). Shake flasks were inoculated with a 10-ml culture seeded with the glycerol stock solutions and grown overnight in 50-ml tubes. Growths were performed in three independent replicates with initial *Abs*_600_ between 0.09 and 0.1, and stopped at different time points (1, 3, 5, 8, 16 and 24 h). After the growth, the broth was centrifuged at 13000 rpm for 40 min (Avanti J20-XP, Beckman Coulter, U.S.A.) and the biomass was separated from the supernatant. One millilitre of supernatant was ultra-filtered on 10 kDa filter devices (Centricon, Amicon, U.S.A.) at 4°C and 13000 rpm (Z216 MK, Hermle Labortechnik GmbH, Germany), the retentate volumes were used to determine the K4 CPS or the K5 CPS released in the growth medium by capillary electrophoresis, according to a previously described method [25], whereas the permeate volumes were used to determine the residual glycerol content and, in case, to determine the added monosaccharide concentrations at different time points of the growths, according to previously described methods [5,14]. The UDP-sugars were extracted from the cells by modifying a previously reported protocol [26]: the pre-cooled biomass (at 4°C) was suspended in a cold methanol–water (1:1 v/v) solution, in a ratio of 6.2 ml for each 0.52 g_cdw_, the suspension was warmed up for 30 min at 70°C and 300 rpm in a water bath and then centrifuged at 4°C and 6000 rpm for 30 min to remove the cell debris. The extracted supernatants (1 ml) were then concentrated up to 20-fold in a vacuum centrifuge (5415 R, Eppendorf, U.S.A.) to determine the UDP-sugar content and the inner CPS one by capillary electrophoresis.

### High-performance capillary electrophoresis method development and analyses

Capillary electrophoresis analyses were performed by using an HPCE instrument (P/ACE MDQ; Beckman Coulter, U.S.A.), equipped with a deuterium lamp and a photodiode array detector, in an uncoated fused-silica capillary (50 μm I.D., 70 cm of total length, 60 cm of effective length; Beckman Coulter, U.S.A.). The analytical conditions for UDP-sugar separation were optimized by using an equimolar mixture solution of the four nucleotide sugar standards (0.4 mM each) and two different running buffers [Buffer 1: 60 mM SDS, 50 mM disodium hydrogen phosphate, 20 mM sodium tetraborate; Buffer 2: 90 mM SDS, 50 mM disodium hydrogen phosphate, 7 mM sodium tetraborate; each one buffered at pH 9.0 with 1 M HCl, degassed and filtered through a 0.45 μm membrane (Millipore, France), before use]. Before each analysis, three 5-min pre-run rinses at 40 psi with 0.3 M NaOH with double-distilled water and then with the running buffer were executed. Sixty nanolitres of sample were injected automatically in pressure mode at 1.0 psi and the separation was performed in normal polarity mode at 18°C (or at 15°C) and at 15, 18 or 22 kV. After each run, two 5-min rinses with double-distilled water and 0.3 M NaOH were performed in reverse mode at 20 psi. The running buffer was refreshed every three runs to avoid any buffer warming effects on the analyses. The possibility of determining the K4 CPS and K5 CPS inner portion (internal or cell-bound) in the same optimized analytical method was tested by using as standards purified polysaccharides obtained by batch fermentation and following purification by ion-exchange chromatography, as already described [25]. Because previous data reported that in shake flask conditions also a defructosylated K4 CPS form (D-K4 CPS) is produced by *E. coli* K4 [5], also the D-K4 CPS standard was prepared as previously reported [25 ] and analysed by capillary electrophoresis in the optimised operative conditions. To evaluate the maximum UV absorbance of the analytes the UDP-sugars and the CPSs were detected in a range from 190 to 300 nm. Peak areas were determined by using the Beckman Coulter 32 Karat Software (Beckman Coulter, U.S.A.). The reproducibility of all the analyte migration times (*t*_migr_) and of their electrophoretic mobilities (μ_ep_) was determined by injecting six times the standard solutions. Linearity ranges and method sensitivity in terms of the lowest detection limit (LOD as a signal-to-noise ratio (S/N) higher than 3) and in terms of the lowest quantification limit (LOQ) were determined by using solutions of the four UDP-sugar standards in the range from 10 to 460 μM and in the range from 0.1 to 2.0 ng/nl for the CPSs; each solution was injected three times and the calibration curves for each analyte were calculated by plotting the averaged areas versus the different concentrations. The optimized method was then used to analyse triplicate samples of the extracted intracellular UDP-sugar concentrations whose values, determined in micromolarity, were then normalized for the cellular biomass by dividing them for the cell dry weight. Accuracy of the method was evaluated by running in triplicate the UDP-sugar extracted samples of the fifth hour from *E. coli* K4 and K5 growths with and without the spiking of UDP-Glc and UDP-GlcA standard solution, which had the highest and the lowest concentrations in the samples respectively. Spiked solutions were prepared adding the standards over a range of 50–150% of the concentrations detected in the samples. Precision of the method was determined as run-to-run repeatability by injecting consecutively three times the extracted samples of each time point of the growth that were run in triplicate in shake flasks (thus 3 × 3 for each time point) and as day-to-day reproducibility by injecting consecutively the samples on two different days. Contemporary also the K4 or K5 CPS content, extracted of the *E. coli* K4 or K5 biomasses at different time points of shake flask growths, was determined (in case of presence of D-K4 CPS, the contribution of this peak on the inner total CPS content was taken in account). The concentrations of the K4 and K5 CPS released in the culture medium during the growths were determined, instead, by capillary electrophoresis according to a previously described method [25] and the total concentration of the produced CPSs, at different time points of the growth, was calculated as the sum of the inner and outer ones.

## Results

### Method development

**Figure 2:**
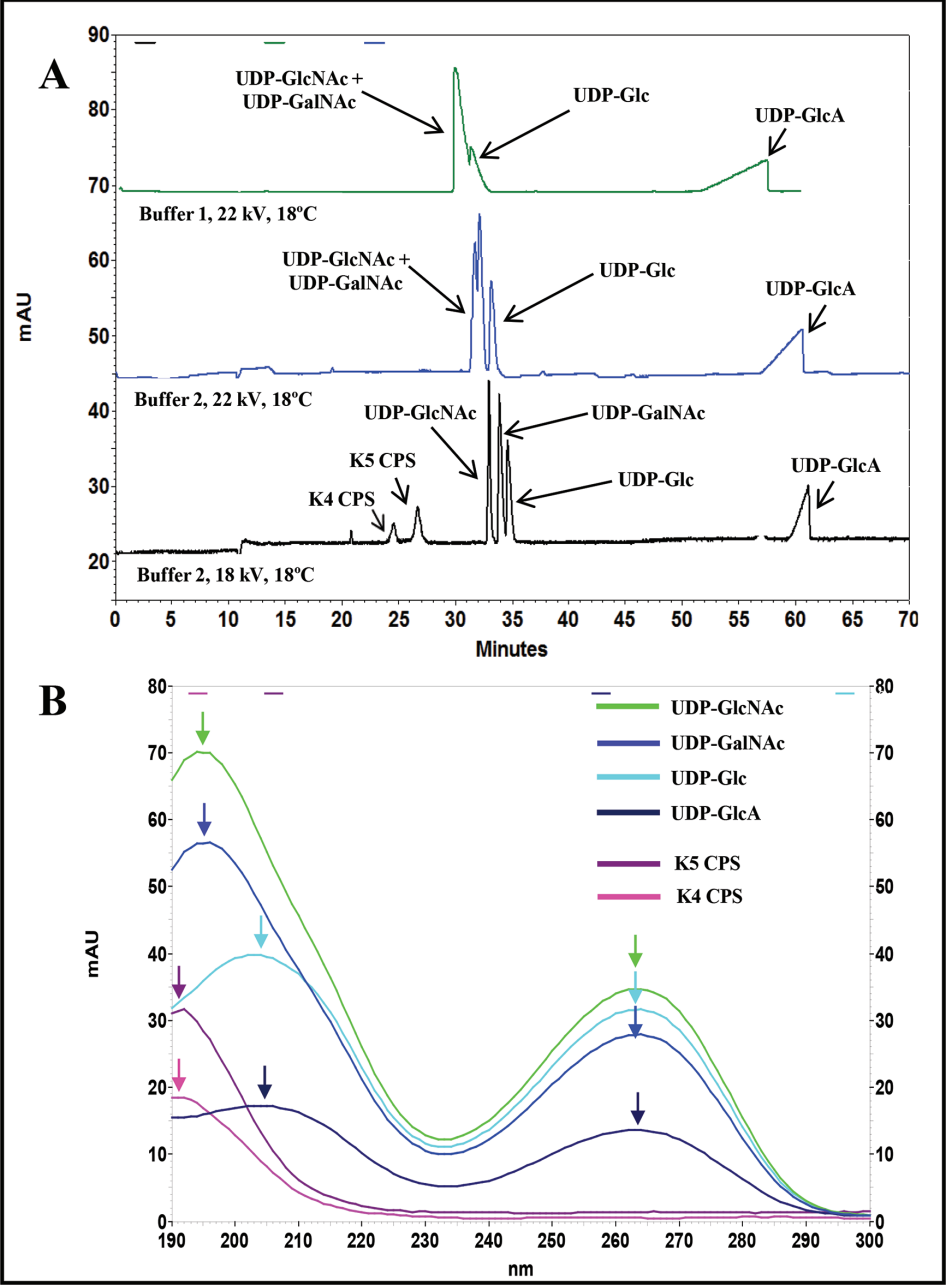
HPCE separation of the UDP–sugar mixture in different operative conditions, with Buffer 1, at 22 kV and 18°C; with Buffer 2, at 22 kV and 18°C; and contemporary HPCE separation of the UDP–sugar mixture and the K4 and K5 CPSs with Buffer 2, at 18 kV and 18°C (A) Overlaid absorbance spectra of the UDP-sugars, of the K4 and the K5 CPSs in the range from 190 to 300 nm with the highest absorbance maximum values indicated by the arrows (B).

Different analytical conditions were explored to obtain the contemporary separation of UDP-GlcNAc, UDP-GalNAc, UDP-Glc and UDP-GlcA by HPCE. Using the same buffer and operative conditions reported in a previous study [18] (Buffer 1 at 22 kV and 18°C, see ‘Materials and methods’), no separation of UDP-GlcNAc and UDP-GalNAc occurred (Figure 2A). By modifying the buffer composition, an initial separation of UDP-GlcNAc, UDP-GalNAc and UDP-Glc was noted but the peaks resulted completely separated only after changing also the voltage setting at 18 kV (Figure 2A). In these new operative conditions, all the UDP-sugars showed distinctive migration times (Table 1) with separation of 0.70 ± 0.05 min difference between UDP-GlcNAc and UDP-GalNAc and of 0.90 ± 0.03 min difference between UDP-GalNAc and UDP-Glc (Table 1); besides in these conditions, the UDP-GlcA peak shape improved and resulted narrower with a width reduced of 80%. A reduction of the potential used in the method (15 kV) did not help to further increase the peak separation between UDP-GlcNAc and UDP-GalNAc and UDP-Glc, on the contrary a temperature diminishing (15°C) improved the peak separation (0.76 ± 0.03 min difference between UDP-GlcNAc and UDP-GalNAc and 1.01 ± 0.02 min difference between UDP-GalNAc and UDP-Glc respectively) but it prolonged the running time up to 100 min (results not shown). The method was also able to separate the K4 and the K5 CPSs that migrated with two distinctive times and electrophoretic mobility values, different from the four UDP-sugar ones (Figure 2A; Table 1). Because it was previously demonstrated that *E. coli* K4, grown in shake flasks, could produce both the K4 CPS and its defructosylated form (D-K4 CPS) [5], we tested the possibility to separate the two species with this method. The D-K4 CPS peak was separated from the K4 CPS and, as expected, it showed the same migration time of the K5 CPS (Table 1). The maximum absorbance of the analytes was also investigated: all the UDP-sugars showed a first characteristic, specific maximum at 262 nm, wavelength that is traditionally reported in literature for their detection [23,24]. But a second one, of higher intensity, was noted at 195 nm for UDP-GlcNAc and UDP-GalNAc, or at 205 nm for UDP-Glc and UDP-GlcA (Figure 2B), thus an intermediate value at 200 nm was selected as the most suitable wavelength for the contemporary detection of all the four species. At 200 nm the peak areas of the nucleotide sugars resulted always higher than the ones observed at 262 nm (the averaged area values were higher of 47.8 ± 5.9% for UDP-GlcNAc, of 45.7 ± 2.5% for UDP-GalNAc, of 25.5 ± 1.7% for UDP-Glc and of 39.3 ± 1.3% for UDP-GlcA). The CPSs showed, as expected, a maximum absorbance at 190 nm, but their determination has been already previously reported at 200 nm as well (Figure 2B) [25]. The method reproducibility of both migration times and electrophoretic mobility was also tested for all the six molecules: the four UDP-sugars as well as the two CPSs showed low standard deviations and coefficient of variation values (Table 1). The method linearity was evaluated too: the calibration curves of the UDP-sugars showed a good linearity in the range from 10 to 460 μM (corresponding to an amount of injected sample from 0.9 to 18 ng) with correlation factors above 0.990 and a limit of detection of 5 μM (Table 1 and Supplementary Figure S1A). Low percentage deviation values of the peak areas (from 4.3 to 6.3%) were obtained when the UDP-sugar standard solutions were analysed consecutively in triplicate. The nucleotide solutions remained stable for 2 months if stored at –20°C (area standard deviation values ranged from 2.0 to 5.4%); after 3 months of storage, the UDP-GlcA solutions started to degrade and standard deviation reached values of 12.4%. Calibration curves of the three CPSs showed linearity in the range from 0.1 to 2.0 ng/nl with correlation factors higher than 0.990 and a limit of detection of 0.03 ng/nl (Table 1 and Supplementary Figure S1B).

**Table 1:** Migration times and electrophoretic mobility values (with the standard deviations and the percentages of coefficient of variation), linearity ranges and correlation factors, sensitivity limits (as LOD and LOQ) of UDP-GlcNAc, UDP-GalNAc, UDP-Glc, UDP-Gal, UDP-GlcA, K4 CPS, K5 CPS and D-K4 CPS, separated by HPCE at 18 kV, 18°C, by using Buffer 2

Analyte	Migration time			Electrophoretic mobility			Linearity		Sensitivity	
	*t*_migr_ (min)	*t*_migr_ S.D. (min)	CV (%)	μ_ep_ (cm^2^ ·V^−1^ ·sec^−1^)*10^−5^	μ_ep_ S.D. (cm^2^ ·V^−1^ ·sec^−1^)*10^−5^	CV (%)	Range (μM)	Correlation factor (*R*^2^)	LOD (μM)	LOQ (μM)
**UDP-GlcNAc**	33.00	0.20	0.60	−2.16	0.01	0.28	10-460	0.998	5	10
**UDP-GalNAc**	34.10	0.32	0.94	−2.19	0.01	0.48	10-460	0.997	5	10
**UDP-Glc**	35.00	0.25	0.73	−2.21	0.01	0.22	10-460	0.997	5	10
**UDP-Gal**	35.55	0.06	0.16	−2.24	0.01	0.06	10-460	0.998	5	10
**UDP-GlcA**	60.71	0.59	0.95	−2.65	0.01	0.10	10-460	0.992	5	10
	***t*_migr_ (min)**	***t*_migr_ S.D. (min)**	**CV (%)**	μ_ep_ (cm^2^ ·V^−1^ ·sec^−1^)*10^−5^	μ_ep_ S.D. (cm^2^ ·V^−1^ ·sec^−1^)*10^−5^	**CV (%)**	**Range (ng/nl)**	**Correlation factor (*R*^2^)**	**LOD (ng/nl)**	**LOQ (ng/nl)**
**K4 CPS**	24.20	0.10	0.70	−1.80	0.01	0.79	0.1-2.0	0.990	0.03	0.10
**D-K4 CPS**	27.12	0.20	0.8	−1.94	0.01	0.86	0.1-2.0	0.993	0.03	0.10
**K5 CPS**	27.12	0.20	0.75	−1.94	0.01	0.49	0.1-2.0	0.994	0.03	0.10

### Analyses of *E. coli* K4 and K5 shake flask growth extracts

**Figure 3:**
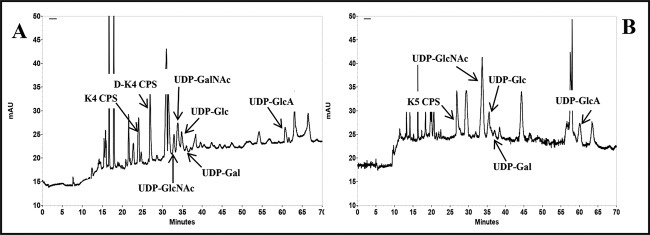
HPCE electropherograms of *E. coli* K4 (A) and K5 (B) UDP-sugars and of the inner K4 and K5 CPSs as extracted from shake flask biomasses

**Figure 4:**
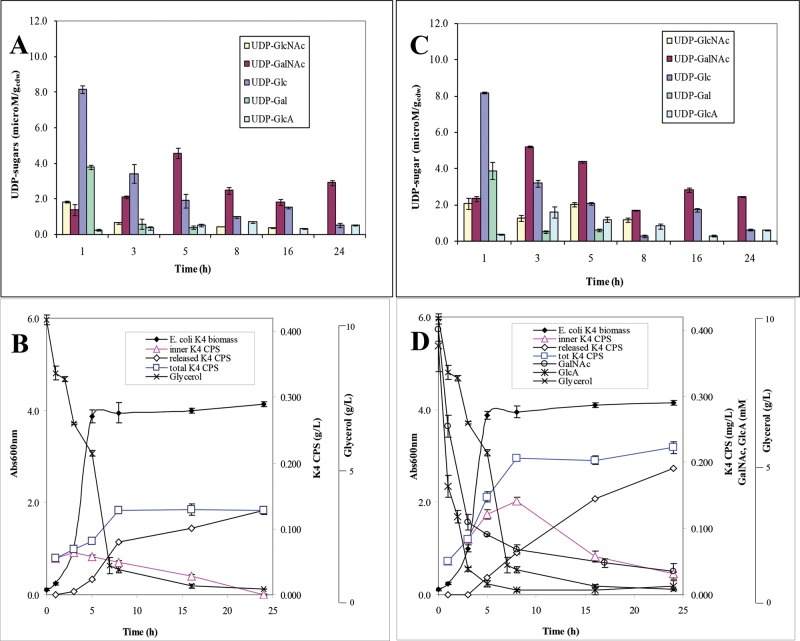
*E. coli* K4 shake flask experiments: UDP-sugar profiles, growth and glycerol consumption curves, released, inner and total K4 CPS production, and eventually the consumption curves of the added monosaccharides, in the glycerol and soya peptone medium (A and B) or in the monosaccharide supplemented one (C and D)

**Figure 5:**
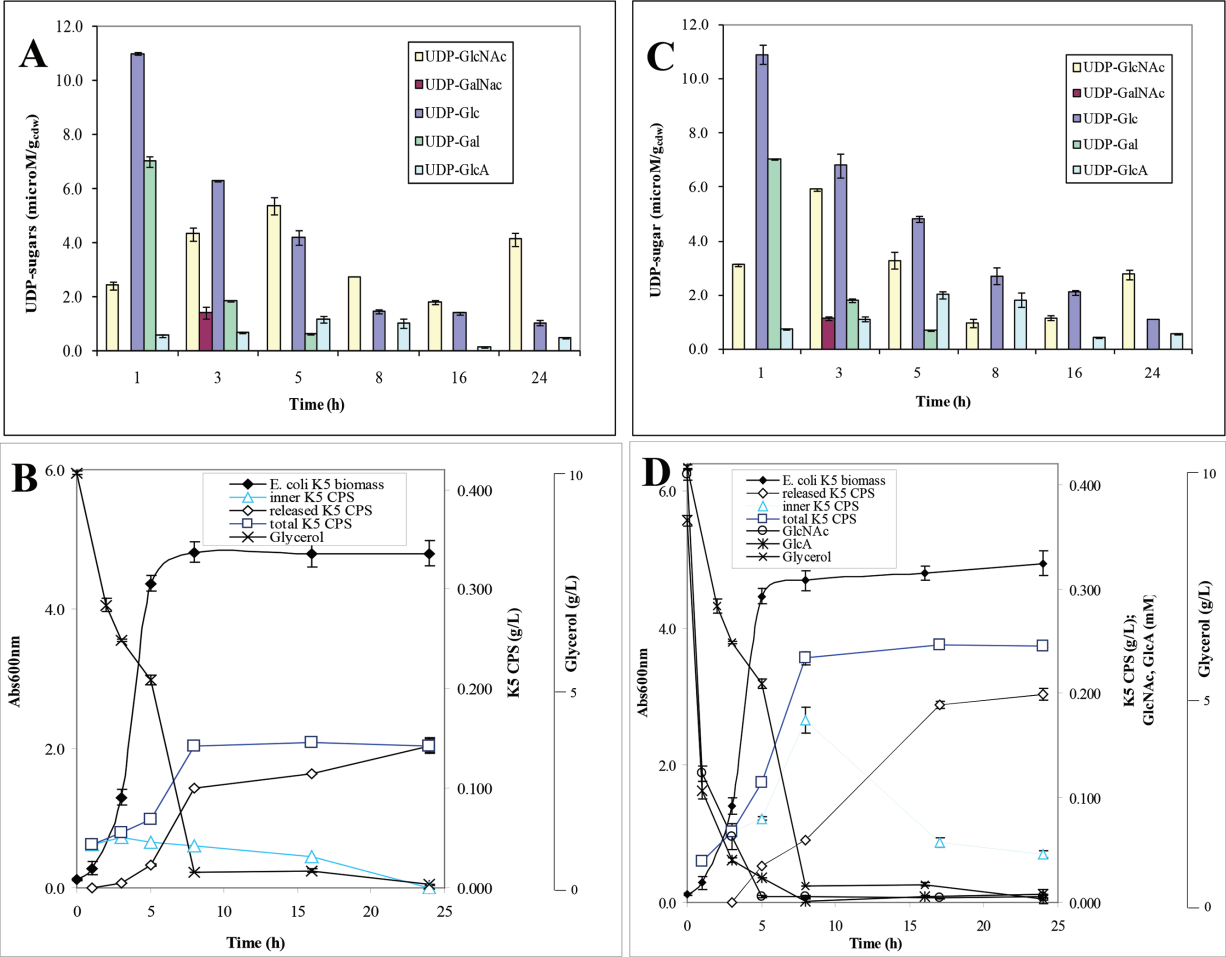
*E. coli* K5 shake flask experiments: UDP-sugar profiles, growth and glycerol consumption curves, released, inner and total K5 CPS production, and eventually the consumption curves of the added monosaccharides, in the glycerol and soya peptone medium (A and B) or in the monosaccharide supplemented one (C and D)

Following the aim to obtain a fingerprint of the nucleotide sugar profile in diverse moments of the bacteria growths, and eventually in different conditions, simultaneously to the determination of the inner CPS production, this method was used to analyse extracts from *E. coli* K4 or K5 bacterial cells at different time points of shake flask growths performed on a glycerol-based medium and on the same medium supplemented with the GalNAc or GlcNAc and GlcA monosaccharide precursors. In the electropherograms of the extracted samples, the nucleotide sugars and the CPSs showed the same migration times of the correspondent standards (deviation values were in the range of 0.6–0.7% for UDP-GlcNAc, UDP-GalNAc and UDP-Glc, 1.1–1.3% for UDP-GlcA and 0.5–0.6% for the CPSs with similar intra- and inter-day variations) (Figure 3A and B) (one step of extraction from biomass was sufficient to recover all the UDP-sugars and the inner CPSs, the samples extracted from the cells in a second or in more following steps did not show any significant peaks). In the extracted samples of both strains, analyses detected also a peak at 35.5 min that resulted to be UDP-Gal (Figure 3A and B). Thus, the analytical method was also able to separate this nucleotide and we decided to include it in our analyses although this UDP-sugar was not directly involved in the pathway for the K4 and K5 CPSs synthesis. A calibration curve of this nucleotide sugar was built and the method linearity and sensitivity resulted similar to the ones already reported for the other nucleotide sugars (Table 1 and Supplementary Figure S1A). Quantitative analyses determined the intracellular UDP-sugar concentrations with high reproducibility and reliability: accuracy was determined as 98.5% whereas precision as run-to-run repeatability was 0.58% and as day-to-day reproducibility was 0.87%. UDP-sugar concentrations varied between 0.25 and 11.0 μM·g_cdw_^−1^ (from 0.2 to 12.0 μM) according to the type of precursor, the growth phase, the strain and the cultivation medium and their availability really influenced the total CPS production (Figures 4 and 5). In particular, starting analysing data from *E. coli* K4 experiments on the glycerol-based medium, distinctive phases in terms of nucleotide sugar profiles and of capsular production were observed; at the beginning of the exponential phase, when the substrate was quickly consumed and the biomass increased, the concentrations of UDP-GlcNAc, UDP-Glc and also of UDP were higher than the UDP-GalNAc and UDP-GlcA ones, even of 32-fold (Figure 4A and B). Besides, since the beginning, the UDP-GalNAc and UDP-GlcA showed differences in their concentration ranges, with lower values for UDP-GlcA (concentrations changed during the growth from 1.4 to 4.6 μM·g_cdw_^−1^ for UDP-GalNAc and from 0.25 to 0.55 μM·g_cdw_^−1^ for UDP-GlcA). In the exponential phase the UDP-GalNAc and UDP-GlcA amounts increased and reached their maximum at 5 h when their concentrations were correspondently 3.2- and 1.9-fold higher than the initial ones. But they improved with different rates (averaged improvement rates was of 34 and 54% for UDP-GalNAc between 1–3 h and 3–5 h and of 13 and 42% for UDP-GlcA, correspondently) thus that ratio between these two precursors remained unbalance during the whole growth (the UDP-GalNAc/UDP-GlcA ratio was between 5.4 and 8.9) (Figure 4A). In the same phase the other two nucleotides, their precursors, instead, decreased (from 8.1 to 3.0 μM·g_cdw_^−1^ for UDP-Glc and from 1.8 μM·g_cdw_^−1^ to a not detectable peak for UDP-GlcNAc), thus a process of partial conversion of UDP-Glc in UDP-GlcA and of UDP-GlcNAc in UDP-GalNAc might be hypothesized (Figure 4A). Also the UDP-Gal decreased and resulted completely consumed at the third hour of growth. Between the first and the fifth hour, the CPS was mainly found as inner portion and its production increased differently with the highest rate noted between the fifth and the eighth hour after that the UDP-GalNAc and UDP-GlcA reached their maximum (the production improvement averaged values were of 19% between 1 and 3 h, of 14% between 3 and 5 h and of 36% between 5 and 8 h) (Figure 4A and B). At the eighth hour the total CPS production reached its maximum and both UDP-GalNAc and UDP-Glc were consumed (Figure 4A and B). From that point up to the 24 h, with the slowdown of the growth caused by the diminishing of the carbon source in the medium, a small accumulation of UDP-GalNAc and a stable concentration of UDP-GlcA were determined and no further production of CPS was noted but the cells simply released it completely in the surrounding medium (24 h K4 CPS final concentration value was consistent with the one already previously reported for shake flask experiments (Figure 4A and B, Supplementary Figure S2) [5]. Because the low UDP-GalNAc and UDP-GlcA initial concentrations might be a limiting factor in the CPS synthesis, shake flask experiments with supplementation at the beginning of the growth were performed (Figure 4C and D). Previous studies have already established that this kind of supplementation drives to a 68% increased K4 CPS production at 24 h [14], but neither the kinetic of the total CPS production nor the UDP-sugar concentration variations were investigated. The supplementation increased the precursor availability since the first hour of growth (UDP-GalNAc and UDP-GlcA were 1.7- and 1.5-fold higher than in the normal glycerol medium) and their maximum was reached at the third one when the 73% of the supplemented GalNAc and 90% of GlcA were already uptaken from the medium respectively (UDP-GalNAc and UDP-GlcA were 2.4- and 4.4-fold higher than in the normal glycerol medium) (Figure 4C). This caused an increase in the K4 CPS production rate that reached values of 45 and 37% between the first and the third hour and the third and the fifth one, correspondently (increase in 2.3- and 3.1-fold) (Figure 4D). At the fifth hour, the UDP-GlcA was 2.3-fold higher than in normal medium growth whereas the UDP-GalNAc resulted similar. The supplementation was more effective in boosting UDP-GlcA than UDP-GalNAc thus the differences between their concentrations were reduced (UDP-GalNAc/UDP-GlcA ratio between 2.0 and 6.2), whereas it did not influence the UDP-Glc, UDP-Gal and UDP-GlcNAc initial concentrations although this precursor at the fifth hour was almost 1.9 times higher than in the normal glycerol medium, probably because of a diverse process of conversion in UDP-GalNAc, altered by the supplementation (Figure 4C). At the eighth hour all the UDP-sugars resulted diminished whereas the total K4 CPS production reached its maximum; then the capsule was completely released in the medium and its final concentration resulted 2.2-fold higher than the one obtained in the growth on the normal glycerol medium, as previously reported data (Figure 4C and D) [14]. In *E. coli* K5 experiments on the glycerol-based medium, the UDP-sugars varied with trends comparable with the ones observed for *E. coli* K4 but small differences were observed and higher concentrations of all the nucleotides were determined since the beginning of the growth (up to 1.3-fold for UDP-GlcNAc and UDP-Glc, 1.8-fold for UDP-Gal and of 2.3-fold for UDP-GlcA) and thus a narrower UDP-GlcNAc/UDP-GlcA ratio difference was estimated (between 4.1 and 6.0) (Figure 5A) (in this case the UDP-GlcNAc has not the role of precursor of UDP-GalNAc as in *E. coli* K4 and thus its trend resulted similar to the UDP-GalNAc one observed in *E. coli* K4 growths. On the other hand, UDP-GalNAc was detected in *E. coli* K5 extracted samples only at the third hour of growth). The UDP-GlcNAc and UDP-GlcA concentrations increased during the exponential phase reaching their maximum at the fifth hour (averaged improvement rates of 43 and 19% for UDP-GlcNAc between 1–3 h and 3–5 h and of 18 and 45% for UDP-GlcA, correspondently), whereas the UDP-Glc decreased as previously noted (Figure 5A). This higher availability of precursors determined a higher total final K5 CPS concentration, produced with higher rates, with a maximum between the fifth and the eight hour as previously observed for *E. coli* K4 (the production improvement averaged values were of 20% between 1 and 3 h, of 18% between 3 and 5 h and of 50% between 5 and 8 h) (Figure 5B, Supplementary Figure S2). Also in this case the addition of GlcNAc and GlcA in the medium, at the beginning of the growth, drove to a 2-fold increased production rates between the first and the third hour and the third and the fifth one (43 and 41%), thanks to higher inner UDP-GlcNAc and UDP-GlcA concentrations (between 1.2- and 1.4-fold for UDP-GlcNAc and 1.2- and 2.4-fold for UDP-GlcA respectively; UDP-GalNAc/UDP-GlcA ratio between 1.63 and 2.0) (Figure 5C and D). At the fifth and eighth hour, the UDP-GlcNAc was consumed and the total K5 CPS production reached its maximum concentration, a value 1.8-fold higher compared with the normal medium one.

## Discussion

In the last years, new studies demonstrated the possibility to obtain CS and HP by biotechnological processes starting from the bacterial CPSs of *E. coli* K4 and K5 whose structures resemble the chondroitin and the heparosan ones [5–11]. The CPS production changes according to the different growth and metabolic conditions, thus determining the inner concentrations of UDP-sugars and their variations during the bacterial growth and correlating them with the total CPS production, could provide additional information on the synthetic mechanism and its bottlenecks as well as could offer a metabolic fingerprint profile for both wild-type and recombinant strains. No one has reported a similar study before, according to our knowledge: so far only a paper reported the analyses of the UDP concentrations at only one single point during the exponential growth phase of some *Streptococcus* strains producing hyaluronic acid [27]. For these reasons we aimed to develop a new, improved, reliable, specific capillary electrophoresis method for the contemporary analysis of UDP-GlcNAc, UDP-GalNAc, UDP-Glc and UDP-GlcA, as extracted from *E. coli* K4 and K5 cells and that, in addition, could result useful also to detect the inner CPS portion in order to correlate the nucleotide sugar pool variations with the total CPS production. During the method development studies, the separation of three on four UDP-sugars proved to be tricky, due to the analogous migration and running behaviours of UDP-GlcNAc, UDP-GalNAc and UDP-Glc; a complete separation was reached by using a newly formulated buffer and by modifying the voltage conditions. Up to now, the detection of UDP-sugars by capillary electrophoresis was always performed at wavelength values between 255 and 262 nm [23,24]; but in this research, instead, we demonstrated that the four nucleotide sugars showed higher absorbance at 200 nm. In the optimized operative conditions, this method demonstrated to be a good tool to quantitatively determine the four nucleotide sugars at micromolar concentrations, and it showed similar sensitivity, but wider linearity range, than the ones already reported before [23,24]. It also results in the ability to detect and separate a fifth UDP-sugar, the UDP-Gal, which although not directly involved in the K4 and K5 CPSs synthesis, was taken in consideration in our analyses. Applied to the determination of the UDP-sugars extracted from the bacterial biomasses the method allowed to profile, for the first time, the pools of *E. coli* K4 and K5 nucleotide sugars, to determine the intracellular concentrations at different time points of shake flask growths and to detect differences according to the growth phase, the type of precursors, the strain and the medium. The analyses performed on both strains showed a general trend in which the nucleotide sugars directly involved in the polymerization process (like UDP-GalNAc, UDP-GlcNAc and UDP-GlcA) showed low concentrations in the first hours of growth, when the culture quickly duplicated and produced also lower CPS amounts; whereas higher ones were noted in the middle of the exponential phases when the growth slowed down and the most consistent part of the total CPS was produced. That demonstrated the existence of a real process of UDP-sugar formation during the growth and underlined how the production of CPS was strictly dependent on the nucleotide sugar availability. The UDP-sugar values observed at the fifth and at the eighth hours resulted in the same range of the one reported for the nucleotide precursors of different *Streptococcus zooepidemicus* recombinant strains as determined at only one time-point in the middle of the exponential phase in 2-litre fermentations [27]. Although analyses detected always higher nucleotide sugar concentrations in *E. coli* K5 than in *E. coli* K4, in both strains a strong difference between UDP-GalNAc or UDP-GlcNAc and UDP-GlcA amounts was observed during the whole growth demonstrating a different speed in the formation of the precursors and an unbalanced ratio between them. The lower amount of UDP-GlcA could be a limiting factor in the capsular chain assembly [28] as well as it could have a key role in regulating the glycotrasferase activity as hypothesized in previous studies [15,29]. But the method was also able to detect specific differences in the nucleotide sugar pools in case of changes of growth conditions, confirming that the addition of GalNAc or GlcNAc, and GlcA in the medium effectively increased the UDP-GalNAc or UDP-GlcNAc and UDP-GlcA concentrations inside the cells since the beginning of the growth and this drove to boost immediately the inner K4 and K5 CPS production and thus the total final one.

In conclusion, this new capillary electrophoresis method demostrated to be a good analytical tool to determine the UDP-sugar concentrations and the inner CPSs during *E. coli* K4 and K5 growths (but eventually also of other different microbial capsulated strains like the hyaluronic acid producing *Streptococcus* ones), providing information and revealing changes of the nucleotide sugar fingerprint profile according to the diverse growth and metabolic conditions, thus opening the way to better understand and, eventually, to sort out the bottlenecks in the synthetic pathway and to design new, more efficient, tailored-cut metabolic engineering strategies.

## Abbreviations

CPS: capsular polysaccharide
CS: chondroitin sulfate
D-K4 CPS: defructosylated CPS from *Escherichia coli* K4
HP: heparin
HPCE: high-performance capillary electrophoresis
K4 CPS: CPS from *Escherichia coli* K4
K5 CPS: CPS from *Escherichia coli* K5
UDP-Gal: UDP-galactose
UDP-GalNAc: UDP-*N*-acetylgalactosamine
UDP-Glc: UDP-glucose
UDP-GlcA: UDP-glucuronic acid
UDP-GlcNAc: UDP-*N*-aceylglucosamine
